# Impact of aspartate aminotransferase–to–platelet ratio index based score to assess posthepatectomy liver failure in patients with hepatocellular carcninoma

**DOI:** 10.1186/s12957-022-02714-y

**Published:** 2022-08-02

**Authors:** Kyohei Yugawa, Takashi Maeda, Shigeyuki Nagata, Jin Shiraishi, Akihiro Sakai, Shohei Yamaguchi, Kozo Konishi, Kenkichi Hashimoto

**Affiliations:** Department of Surgery, Hiroshima Red Cross Hospital and Atomic-bomb Survivors Hospital, Hiroshima, Japan

**Keywords:** Aspartate aminotransferase-to-platelet ratio index, Posthepatectomy liver failure, Hepatocellular carcinoma

## Abstract

**Background:**

Posthepatectomy liver failure (PHLF) is a life-threatening complication following hepatic resection. The aspartate aminotransferase-to-platelet ratio index (APRI) is a non-invasive model for assessing the liver functional reserve in patients with hepatocellular carcinoma (HCC). This study aimed to establish a scoring model to stratify patients with HCC at risk for PHLF.

**Methods:**

This single-center retrospective study included 451 patients who underwent hepatic resection for HCC between 2004 and 2017. Preoperative factors, including non-invasive liver fibrosis markers and intraoperative factors, were evaluated. The predictive impact for PHLF was evaluated using receiver operating characteristic (ROC) curves of these factors.

**Results:**

Of 451 patients, 30 (6.7%) developed severe PHLF (grade B/C). Multivariate logistic analysis indicated that APRI, model for end-stage liver disease (MELD) score, operating time, and intraoperative blood loss were significantly associated with severe PHLF. A scoring model (over 0–4 points) was calculated using these optimal cutoff values. The area under the ROC curve of the established score for severe PHLF was 0.88, which greatly improved the predictive accuracy compared with these factors alone (*p* < 0.05 for all).

**Conclusions:**

The scoring model-based APRI, MELD score, operating time, and intraoperative blood loss can predict severe PHLF in patients with HCC.

## Introduction

Hepatocellular carcinoma (HCC) is the most common primary liver neoplasm, the sixth most common neoplasm overall, and the third leading cause of death from cancer [1]. Although surgical techniques and perioperative management are safe and effective for patients with HCC, posthepatectomy liver failure (PHLF) remains a fatal postoperative complication [2, 3]. Thus, predictive assessments are important steps in the postoperative management of HCC because severe complications, such as PHLF, depend on the liver functional reserve and degree of fibrosis in individual patients. Therefore, accurate prediction of PHLF is essential in the selection of appropriate and prompt postoperative therapeutic strategies.

Several studies indicated that preoperative and intraoperative factors could help predict surgical outcomes in patients with HCC [4]. In particular, conventional parameters or scores, such as Child–Pugh (C–P) score [5], model for end-stage liver disease (MELD) score [6], albumin–bilirubin (ALBI) grade [6], indocyanine green dye retention rate at 15 min (ICG-R15) [7], operating time, intraoperative blood loss, transfusion, or inflow occlusion time [8], are used widely to assess the risk of postoperative complications. However, it is difficult to predict PHLF with these factors alone.

The liver functional reserve correlates strongly with liver fibrosis or cirrhosis, especially in patients with chronic liver disease. Recently, there has been growing evidence for the utility of non-invasive liver fibrosis-related markers. There are several models for diagnosing liver fibrosis status, such as the platelet-albumin-bilirubin (PALBI) grade [9], fibrosis-4 (FIB-4) index [10], and aspartate aminotransferase (AST)-to-platelet ratio index (APRI) [11, 12]. However, the best predictors of PHLF remain unclear despite accumulating evidence that many of these models correlate to surgical outcomes in patients with HCC.

Previous studies have elucidated a pivotal association of PHLF with each predictive factor and with combinations of these factors in patients with HCC. However, the specific combinations of factors that include non-invasive liver fibrosis are more suitable PHLF predictors than those alone are undetermined in the literature. Our goal was to reveal the predictive utility of a new scoring model of these factors in patients who underwent hepatic resection for HCC.

## Materials and methods

### Patients and ethics

This study included all patients who underwent hepatic resection for primary HCC at Hiroshima Red Cross Hospital and Atomic-bomb Survivors Hospital in Japan between March 2004 and December 2017. Patients received no preoperative chemotherapy or radiation and were selected retrospectively. Anonymized perioperative clinical data of all patients were obtained from electronic and paper records. The ethics committee of our hospital approved this study under the ethical guidelines of the Japanese government (approval number: 2021-029), and all patients provided consent for the use of their clinical data in this research.

### Surgical procedures

Details of the surgical technique and patient selection criteria were described previously [13]. According to the Evidence-based Clinical Practice Guidelines for HCC [14], indications of hepatectomies were good health status with Eastern Cooperative Oncology Group Performance Status of 0–2 and good liver function reserve with Child–Pugh grade A or B and ICG-R15. The underlying liver and tumor status were assessed by abdominal ultrasonography, contrast-enhanced computed tomography (CT), or magnetic resonance imaging. In consideration of the tumor status (such as number, size, location), the decision is made based on whether it was anatomical or non-anatomical hepatectomy. CT volumetry was performed for the assessment of the remnant liver volume. Preoperative liver CT volumetry was mostly performed for patients who received anatomical or major hepatectomy. In addition, patients with severe liver fibrosis or other organ dysfunction were allowed to have a future liver remnant volume at least 40%, and patients with normal liver function were allowed to have a remnant liver volume of > 30%. In nearly all hepatic resections, the intermittent Pringle maneuver was applied, consisting of clamping the portal triad for 15 min and then releasing the clamp for 5-min intervals for hemivascular occlusion.

### Definitions

Major hepatectomy is the removal of 3 or more hepatic segments [15]. On the basis of the International Study Group of Liver Surgery definition [2], patients with increased total bilirubin and international normalized ratio (INR) on day 5 after surgery were considered to have PHLF. In this study, the cutoff values of total bilirubin and INR were defined as 2.9 mg/dL and 1.5, respectively. Patients with PHLF grade A do not require specific therapy, those with grade B require some non-invasive therapies, such as fresh albumin and frozen plasma infusion, and those with grade C require invasive therapies, such as hemodialysis and mechanical ventilation [2]. This study defined PHLF grade B and C patients as severe patients with PHLF.

### Data collection

Preoperative serum samples were collected within 1 week before hepatic resection for HCC. Tumor markers (AFP and DCP) and levels of total bilirubin, albumin, AST, alanine aminotransferase (ALT), creatinine, INR, and platelets were measured before surgery. The C–P score includes total serum bilirubin, prothrombin time, albumin level, and the presence of ascites and hepatic encephalopathy. The MELD score and non-invasive liver fibrosis scores based on these laboratory tests were calculated as previous reports [12].

### Statistical analysis

Data were calculated as means, medians, frequencies, and percentages. We used the Mann–Whitney *U* test and the Kruskal–Wallis test to compare continuous variables. We used the *χ*^2^ test or Fisher’s exact test to compare categorical variables. The receiver operating characteristic (ROC) curves with the Youden’s index correction were estimated to determine the optimal cutoff values for analyzing the risk of PHLF [16]. Comparison of ROC curve analysis was performed by calculating the Standard Error of the area under the curve (AUC), and the differences between two AUCs [17]. Logistic regression analysis was used to perform univariate and multivariate analyses. Variables significant in univariate analyses were selected in the overall multivariate logistic regression model to identify PHLF predictive factors. All statistical tests were two-sided, and a *p* value of < 0.05 indicated significance. All analyses were performed with JMP14pro software (SAS Institute, Cary, NC, USA).

## Results

### Patient characteristics

Table 1 summarizes the baseline characteristics of the 451 patients with HCC who underwent liver resection. The patient population comprised of 146 (32.4%) females and 305 (67.6%) males with a median age of 71 years (range 35–91 years). The causes of HCC included hepatitis B virus infection in 65 (14.4%), hepatitis C virus infection in 280 (62.1%), and liver cirrhosis (F4) in 206 (45.7%) HCC patients. According to the Barcelona Clinical Liver Cancer (BLCL) grading, 109 (24.1%) patients were classified as grade 0, 261 (57.9%) as grade A, and 81 (18.0%) as grade B.

**Table 1:** Baseline characteristics of included 451 patients with HCC and comparison of factors between patients with and without severe PHLF

Variables	All patients (***n*** = 451)	Severe PHLF		***P*** value
		No (***n*** = 421)	Yes (***n*** = 30)	
Age (years)	71 (35–91)	71 (35–91)	68 (35–82)	0.2788
Sex, female/male/female	146/305	139/282	7/23	0.2734
BMI (kg/m^2^)	23.2 (13.4–34.1)	23.3 (13.4–34.1)	21.6 (19.2–29.0)	0.0237*
Etiology HBV/HCV/HBV + HCV/NBNC	65/280/5/101	59/259/5/98	6/21/0/3	0.3103
Albumin (g/dL)	4.0 (2.5–5.1)	4.0 (2.5–5.1)	3.7 (3.0–4.9)	0.0054*
Total bilirubin (mg/dL)	0.7 (0.2–2.0)	0.7 (0.2–2.0)	1.0 (0.4–1.9)	0.0012*
AST (IU/L)	37 (8–252)	36 (8–252)	55 (32–150)	< 0.0001**
ALT (IU/L)	34 (6–312)	33 (6–312)	47 (20–245)	0.0004**
Platelet count (× 10^9^/L)	134 (24–660)	139 (24–530)	89 (33–660)	0.0019*
PT (%)	91.5 (46.3–130.3)	91.9 (46.3–130.3)	82.3 (49.2–107.9)	0.0019*
INR	1.06 (0.88–1.67)	1.05 (0.88–1.67)	1.11 (0.97–1.56)	0.0023*
Creatinine (mg/dL)	0.76 (0.20–7.11)	0.76 (0.2–7.11)	0.84 (0.5–6.0)	0.0152*
Urine nitrogen (mg/dL)	14.8 (5.3–48.9)	14.8 (5.3–48.9)	14.7 (9.3–47.5)	0.6681
ICG-R15 (%)	16.8 (0.9–67.0)	16.5 (0.9–67.0)	24.0 (3.9–54.6)	0.0068*
PALBI	− 2.66 (− 3.43 to − 1.31)	− 2.67 (− 3.43 to − 1.65)	(− 2.45 (− 3.14 to (− 1.31)	0.0055*
APRI	0.96 (0.09–12.7)	0.91 (0.09–12.7)	2.16 (0.19–8.99)	< 0.0001**
FIB-4 index	3.3 (0.5–100.6)	3.2 (0.5–100.6)	6.5 (0.7–22.2)	< 0.0001**
ALPlat index	500 (322–975)	504 (322–932)	466 (331–975)	0.0016*
MELD	7.3 (6.4–15.5)	7.3 (6.4–15.1)	8.9 (6.4–15.5)	0.0005**
Child-Pugh grade A/B	438/13	410/11	28/2	0.1998
Blood loss (mL)	270 (0–3330)	260 (0–2800)	695 (33–3330)	0.0003**
Operating time (min)	214 (60–633)	212 (60–630)	276 (90–633)	0.0014*
Extent of hepatectomy Minor/major	406/45	384/37	22/8	0.0016*
AFP (ng/mL)	11.7 (1.0–93721)	11.2 (1.0–46262.6)	39.3 (2.1–93721)	0.0014*
DCP (mAU/mL)	24 (0.01–109830)	24 (0.01–43253)	42 (0.03–109830)	0.1851
BCLC grading 0/A/B	109/261/81	105/245/71	4/16/10	0.0522
TNM staging I–II/III–IV	213/238	206/215	7/23	0.0114*
Tumor size (cm)	2.4 (0.5–13.0)	2.3 (0.5–13.0)	3.0 (1.5–12.0)	0.0069*
Solitary/multiple	326/125	309/112	17/13	0.0479*
Poorly differentiation	114 (25.3%)	105 (24.9%)	9 (30.0%)	0.5379
Microscopic vascular invasion	35 (7.8%)	34 (8.1%)	1 (3.3%)	0.3482
Microscopic intrahepatic metastasis	63 (14.0)	55 (13.1%)	8 (26.7%)	0.0379*
Liver cirrhosis (F4)	206 (45.7%)	187 (44.4%)	19 (63.3%)	0.0445*

Data are presented as *N* or median (range). *AFP* alpha-fetoprotein, *ALPlat index* platelet count + 90 × albumin, *ALT* alanine aminotransferase, *APRI* AST-to-platelet ratio index, *AST* asparate aminotransferase, *BCLC* Barcelona Clinic Liver Cancer, *BMI* body mass index, *CRP* C-reactive protein, *DCP* des-γ-carboxyprothrombin, *FIB-4* fibrosis-4, *HBV* hepatitis B virus, *HCV* hepatitis C virus, *ICG-R15* indocyanine green dye retention rate at 15 min, *MELD* model for end-stage liver disease, *PALBI* platelet-albumin-bilirubin, *PHLF* posthepatectomy liver failure, *PT* prothrombin time, *TNM* tumor, node, metastasis *P < 0.05 and **P < 0.001

Based on the C–P grade, 438 (97.1%) patients were classified as grade A and 13 (2.9%) as grade B. The median MELD score was 7.3 (range 6.4–15.5). Regarding liver fibrosis-related models, the median FIB-4 index was 3.3 (range 0.5–100.6), the median PALBI was − 2.66 (range 3.43 − 1.31), and the median APRI was 0.96 (range 0.09–12.7). Recently, Yamamoto et al. reported that the ALPlat index is useful to predict PHLF [18]. In our cohort, the median ALPlat index was 500 (range 322–975). Regarding operative factors, 45 (10.0%) patients underwent major hepatectomy. The median amount of blood loss was 270 ml (range 0–3330 ml). The median operating time was 214 min (range 60–633 min).

### Comparison of characteristics between patients with and without severe PHLF

Of a total of 451 patients, 74 (16.4%) developed PHLF with 44 (9.8%) classified as grade A, 24 (5.3%) as grade B, and 6 (1.3%) as grade C, while a total of 30 (6.7%) patients were classified to have developed severe PHLF. Clinicopathological characteristics were compared between patients with and without severe PHLF. The values of ICG-R15 (*p* = 0.0068) and MELD score (*p* = 0.0005) were significantly higher in patients with severe PHLF than those without severe PHLF. Regarding non-invasive liver fibrosis markers, all models, such as FIB-4 index (*p* < 0.0001), PALBI (*p* = 0.0055), and APRI (*p* < 0.0001), were significantly higher in the severe PHLF group than the non-severe PHLF group. The ALPlat index was significantly lower in patients with severe PHLF than in those without severe PHLF (*p* = 0.0016). In addition, patients with severe PHLF had a higher incidence of prolonged operating time (*p* = 0.0014) and a higher amount of blood loss (*p* = 0.0003) than those without severe PHLF. Furthermore, in those who underwent major hepatectomy (*p* = 0.0016), the tumor size and number were greater (*p* = 0.0069 and *p* = 0.0479, respectively) than those without severe PHLF (Table 1).

### Independent predictors of severe PHLF

According to the univariate analysis, the significant predictive factors of severe PHLF were higher ICG-R15, MELD score, PALBI, and APRI and lower ALPlat index. Regarding intraoperative factors, prolonged operating time and increased intraoperative blood loss were significant predictive factors for severe PHLF. In the multivariate analysis, higher APRI (odds ratio [OR] 1.32, 95% confidence interval [CI] 1.05–1.65, *p* = 0.0230), higher MELD score (OR 1.44, 95% CI 1.15–1.80, *p* = 0.0020), prolonged operating time (OR 1.01, 95% CI 1.00–1.01, *p* = 0.0019), and remarkable amount of blood loss (OR 1.00, 95% CI 1.00–1.00, *p* = 0.0278) were significant predictive factors of severe PHLF in patients with HCC (Table 2). All predictive factors were set as continuous variables changed by 1 point.

**Table 2:** Univariate and multivariate analyses to identified factors predicting severe PHLF

Variables	Odds ratio	95% CI	***P*** value	Odds ratio	95% CI	***P*** value
PALBI	6.98	2.07–23.6	0.0021*	3.81	0.65–22.3	0.1385
FIB-4 index	1.03	0.99–1.06	0.1674			
APRI	1.36	1.15–1.62	0.0009**	1.32	1.05–1.65	0.0230*
ALPlat index	0.99	0.98–0.99	0.0106*	0.99	0.99–1.00	0.9852
ICG-R15 (%)	1.05	1.01–1.08	0.0070*	1.02	0.97–1.06	0.4890
MELD score	1.43	1.221.68	< 0.0001**	1.44	1.15–1.80	0.0020*
Operating time (min)	1.01	1.00–1.01	< 0.0001**	1.01	1.00–1.01	0.0019*
Intraoperative blood loss (mL)	1.00	1.00–1.00	< 0.0001**	1.00	1.00–1.00	0.0278*

*ALPlat index* platelet count + 90 × albumin, *APRI* AST-to-platelet ratio index, *AST* asparate aminotransferase, *CI* confidence interval, *FIB-4* fibrosis-4, *ICG-R15* indocyanine green dye retention rate at 15 min, *MELD* model for end-stage liver disease, *PALBI* platelet-albumin-bilirubin, *PHLF* posthepatectomy liver failure *P < 0.05 and **P < 0.001

### Predictive performance of the models for severe PHLF

**Fig. 1:**
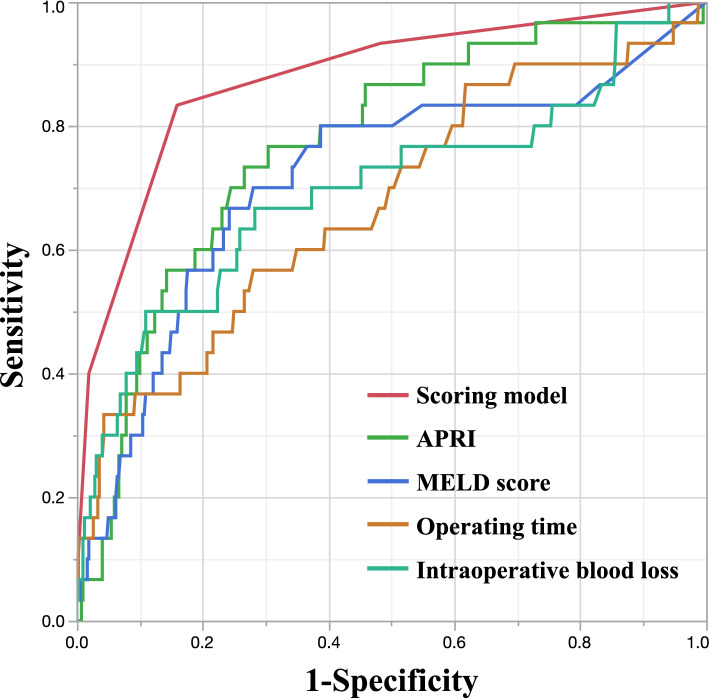
Receiver operating characteristic curves for established scoring model, APRI, MELD score, operating time, and intraoperative blood loss in predicting severe PHLF. APRI, aspartate aminotransferase–to–platelet ratio index; MELD, model for end-stage liver disease

Table 3 summarizes the ROC curve analysis of each model in patients with HCC. Of the preoperative factors, the AUC of APRI was 0.77 (cutoff value 1.56, sensitivity 73.3%, specificity 73.4%, *p* = 0.0009) and that of MELD score was 0.71 (cutoff value 8.3, sensitivity 66.7%, specificity 75.8%, *p* < 0.0001). Of the intraoperative factors, the AUC of the operating time was 0.67 (cutoff value 407 min, sensitivity 33.3%, specificity 95.7%, *p* < 0.0001) and that of the blood loss was 0.70 (cutoff value 847 mL, sensitivity 50.0%, specificity 89.1%, *p* < 0.0001). These values established a new scoring model for each patient as follows: APRI, ≥ 1.56; MELD score, ≥ 8.3; operating time, ≥ 407 min; blood loss, ≥ 847 mL were scored as 1 point each (Table 4). The established score predicted severe PHLF with the highest degree of accuracy compared with the other models (AUC 0.88, sensitivity 83.3%, specificity 84.1%, *p* < 0.0001; Fig. 1). The statistical differences in AUC values between the score and the other models were evaluated. The AUC was significantly higher for the score than for other models in patients with HCC (Table 5).

**Table 3:** Receiver operating characteristic (ROC) curve analysis to evaluate the predictive value of APRI, MELD score, operation time, and blood loss for severe PHLF in HCC patients

Variables	Cut-off	AUC	Sensitivity	Specificity	***P*** value
APRI	1.56	0.76932	73.3	73.4	0.0009**
MELD score	8.3	0.71469	66.7	75.8	< .0001**
Operating time (min)	407	0.67407	33.3	95.7	< .0001**
Intraoperative blood loss (mL)	847	0.69759	50.0	89.1	< .0001**

*APRI* AST-to-platelet ratio index, *AST* asparate aminotransferase, *AUC* area under the ROC curve, *MELD* model for end-stage liver disease; PHLF, posthepatectomy liver failure *P < 0.05 and **P < 0.001

**Table 4:** Selected predictor valiables for multivariable model of severe PHLF in patients with HCC

Variables	No. of risk point for severe PHLF	Odds ratio (95% CI)	***P*** value
APRI			0.0070*
< 1.56	0	1 [Reference]	
≥ 1.56	1	5.17 (2.00–13.4)	
MELD score			0.0001**
< 8.3	0	1 [Reference]	
≥ 8.3	1	9.11 (2.94–28.2)	
Operating time (min)			< 0.0001**
< 407	0	1 [Reference]	
≥ 407	1	16.8 (4.20–67.3)	
Intraoperative blood loss (mL)			0.0056*
< 847	0	1 [Reference]	
≥ 847	1	3.92 (1.49–10.3)	

*APRI* AST-to-platelet ratio index, *AST* asparate aminotransferase, *CI* confidence interval, *MELD* model for end-stage liver disease, *PHLF* posthepatectomy liver failure *P < 0.05 and **P < 0.001

**Table 5:** Comparison of receiver operating characteristic curve analysis to evaluate the predictive value of APRI, MELD score, operating time and intraoperative blood loss for severe PHLF in HCC patients. All statistical tests were two-sided

Variable	AUC	95% CI	***P*** value
Scoring model	0.877	0.79–0.93	Reference
APRI	0.769	0.67–0.85	0.0014*
MELD score	0.715	0.59–0.81	0.0112*
Operating time	0.674	0.56–0.77	0.0006**
Intraoperative blood loss	0.698	0.57–0.80	0.0009**

*APRI* asparate aminotransferase-to-platelet ratio index, *AUC* area under the ROC curve, *CI* confidence interval, *MELD* model for end-stage liver disease, *PHLF* posthepatectomy liver failure *P < 0.05 and **P < 0.001

### Performance of risk stratification based on the PHLF risk score

The cutoff values were determined by ranking patients based on total points and then dividing the patients into three categories (0 point as low risk, 1 to 2 points as medium risk, 3 to 4 points as high risk). The relative risk of severe PHLF in the high-risk group was higher than in the medium- and low-risk groups. Estimated risk rates of the high-risk group were 12.0 to 16.0%, those of the medium-risk group were 0.25 to 4.03, and that of the low-risk group were 0.08 (Table 6).

**Table 6:** Estimated risk rates of stratified category according to risk points for severe PHLF

Severe PHLF risk category	No. of risk points for severe PHLF	Estimated risk of severe PHLF, % (95% CI)	No. with severe PHLF/total no. of patients (%)
Low risk	0	0.08 (0.02–0.31)	2/220 (0.9)
Medium risk	1	0.25 (0.08–0.81)	3/139 (2.2)
	2	4.03 (2.05–7.92)	13/72 (18.1)
High risk	3	12.0 (6.63–21.8)	10/18 (55.6)
	4	16.0 (11.2–23.0)	2/2 (100)

*CI* confidence interval, *PHLF* posthepatectomy liver failure

## Discussion

This study performed logistic regression analysis to evaluate which variables were independent risk factors of severe PHLF in patients with HCC who underwent curative hepatic resection. First, we confirmed the accuracy of APRI, MELD score, operating time, and intraoperative blood loss in predicting severe PHLF using ROC analyses. Second, the developed risk score—the combination of APRI, MELD score, operating time, and intraoperative blood loss—was a more reliable predictor of severe PHLF than other models alone. Finally, we verified that severe PHLF increased along with the risk stratification based on the established risk scoring model.

The non-invasive evaluations of PALBI grade [9], FIB-4 index [19], and APRI [20] were each reportedly associated with the degree of liver fibrosis. However, there is debate on which model is the best predictive factor for PHLF. Our results indicated that APRI—the ratio of platelet count to AST level—was the best independent predictor of severe PHLF in patients with HCC out of all the liver fibrosis models. The platelet count is an important factor in representing liver fibrosis. A low platelet level is associated with advanced liver fibrosis and cirrhosis, as previously reported [21]. In addition, one possible explanation for this observation might be related to the degree of liver damage reflected in the increase of AST, components of the APRI. The serum AST sensitively reflects the presence of liver fibrosis or cirrhosis, the mechanism of which is proposed to be the interruption of clearance of AST and impairment of the mitochondria [22]. It is thus plausible that the APRI can predict severe PHLF more accurately than other models besides PALBI and the FIB-4 index.

Conventional liver functional reserve models, including C-P grade, ICG-R15, and MELD score, are well-known to reflect the function of the liver in patients with chronic liver disease [23]. The C–P grade includes subjective and non-numerical criteria, such as ascites and encephalopathy. Therefore, the C–P grade is not a useful predictor of early postoperative outcomes. The evaluation of ICG-R15 is common in the Eastern population, and its result contributes to minimizing PHLF and mortality following liver resection [24]. The stratification according to ICG clearance has been useful to determine the extent of hepatectomy patients need. Therefore, in patients with high ICG-R15, minimally invasive hepatectomies that can preserve remnant liver function have been selected. That is considered a reason ICG-R15 was not identified as an independent risk factor of severe PHLF. The MELD score is also recognized as a predictor of prognosis in chronic liver disease and is relevant in the early prediction of morbidity and mortality after liver resection [25]. Consistent with the previous reports, our results showed that the MELD score had relatively high accuracy for predicting severe PHLF. However, individual factors such as age and gender can affect serum creatinine levels, which limits its clinical value.

Intraoperative factors, such as operating time and intraoperative blood loss, were included in the logistic regression analysis. Previously, it has been reported that the vascular occlusive techniques and intraoperative blood loss have been known to induce ischemia that can cause reversible or irreversible damage to hepatocytes [26–28]. Thus, it is plausible that operating time and intraoperative blood loss were independent PHLF risk factors in our cohort. These may be associated with systemic hypoperfusion, impaired oxygen delivery to vital organs, and the subsequent suppressed immune response associated with hepatic regeneration [29]. In this study, some tumor factors (high level of AFP, large tumor size, multiple tumors, and intrahepatic metastasis) showed significant differences between patients with severe PHLF and without severe PHLF. This can be explained by the need to expand the liver resection anatomically to the limit of the liver functional reserve as much as possible to prevent positive margins and intrahepatic metastasis.

As mentioned previously, APRI and MELD scores (as preoperative factors) and operating time and intraoperative blood loss (as intraoperative factors) were superior predictors of severe PHLF in patients with HCC. In the clinical setting, we often experience some cases where predicting PHLF only using preoperative factors is difficult. The isolated use of APRI and MELD scores had low discriminatory ability in evaluating liver function accurately because a relatively large number of patients had normal liver function even with advanced fibrosis or cirrhosis. Therefore, even if the preoperative evaluations, including the MELD score, Child–Pugh grade, and ICG-R15, showed satisfactory liver functional reserve, PHLF can occur according to the intraoperative findings, which is one of the reasons why we included the intraoperative factors in our analyses. Accordingly, we developed the APRI–MELD–operating time–intraoperative blood loss scoring model in a simple method by assigning points derived from established cutoff values for a more effective prediction of PHLF. Our scoring model will contribute to the development of appropriate and prompt postoperative therapeutic strategies in patients with HCC.

This study has some limitations. First, the relationship between preoperative liver volume and resected liver weight is not evaluated. Second, most patients with HCC enrolled in this cohort mainly suffered from HCV; therefore, further investigation suitable for each etiological population is required. Finally, this is a proposed study of a new scoring model for predicting severe PHLF. The utility of such new models needs to be demonstrated by internal or external validation. Our established new scoring model for assessing risk of PHLF might not have been completely accurate, because this study lacks validated analyses for the prediction of PHLF. However, this study is considered to have an important message that can be linked to the next study. Further studies are required for a larger population including a validation cohort using an external independent cohort to confirm the validity of the PHLF scoring model in patients who underwent curative surgery for HCC.

## Conclusions

The scoring model including APRI, MELD score, operating time, and intraoperative blood loss can help increase the accuracy of predicting severe PHLF and assess risk stratification. The risk score was more useful for predicting severe PHLF than these models alone, which might help clinicians make informed decisions about treatment and postoperative management strategies in patients with HCC.

## Data Availability

The raw data of this manuscript are available upon reasonable request from the corresponding author.

## Abbreviations

AFP: Alpha–fetoprotein
ALBI: Albumin–bilirubin (score)
ALT: Alanine aminotransferase
APRI: Aspartate aminotransferase–to–platelet ratio index
AST: Aspartate aminotransferase
AUC: Area under the curve
CONUT: Controlling nutritional status
CRP: C-reactive protein
CI: Confidence interval
DCP: Des-gamma-carboxy prothrombin
FIB-4: Fibrosis-4 (index)
HCC: Hepatocellular carcinoma
ICG-R15: Indocyanine green dye retention rate at 15 min
MELD: Model for end-stage liver disease
PALBI: Platelet–albumin–bilirubin (score)
PHLF: Posthepatectomy liver failure
ROC: Receiver operating characteristic
